# Recent Development in Pulmonary Valve Replacement after Tetralogy of Fallot Repair: The Emergence of Hybrid Approaches

**DOI:** 10.3389/fsurg.2015.00022

**Published:** 2015-06-02

**Authors:** Tariq Suleiman, Clifford J. Kavinsky, Clare Skerritt, Damien Kenny, Michael N. Ilbawi, Massimo Caputo

**Affiliations:** ^1^Rush University Medical Center, Chicago, IL, USA; ^2^Bristol Royal Hospital for Children, University of Bristol, Bristol, UK; ^3^Bristol Heart Institute, University of Bristol, Bristol, UK

**Keywords:** pulmonary valve insufficiency, tetralogy of fallot, hybrid intervention, congenital heart disease, cardiac surgery

## Abstract

An increasing number of patients with tetralogy of Fallot require repeat surgical intervention for pulmonary valve replacement secondary to pulmonary regurgitation. Catheter-based interventions have emerged as an attractive alternative to surgery in this patient population but it is limited by patient size or the anatomy of the right ventricular outflow tract. Hybrid approaches involving both cardiac interventionists and surgeons are being developed to overcome these limitations. The purpose of this review is to highlight the recent advances in the hybrid field of pulmonary valve replacement, summarizing the advantages and disadvantages of the “traditional” surgical and the new catheter-based techniques and discuss the direction future research should take to determine the optimal management for individual patients.

## Introduction

In the current era, ~90% of infants born with tetralogy of Fallot (ToF) are expected to live beyond 40 years of age making it the fastest growing population among patients with congenital heart disease (1). One of the most common late consequences after repair of ToF is pulmonary valve regurgitation (PVR). Significant PVR results in progressive dilatation and dysfunction of the right ventricle, decrease in exercise tolerance, arrhythmias, heart failure, and increased risk of sudden death (2). The number of operations for PVR has been increasing over the past decade (3).

The conventional approach of dealing with this problem is to perform pulmonary valve replacement using cardiopulmonary bypass (CPB) and cardioplegic arrest (2). However, this approach is associated not only with long operative times but also side effects related to the use of CPB.

Development of percutaneous approaches to valve disease is one of the most exciting areas of clinical innovation and cardiovascular medicine. The main development has been that of transcatheter pulmonary valve replacement for the rehabilitation of conduits between the right ventricle and pulmonary artery in patients after surgery for ToF. Percutaneous approaches avoid the need for CPB; however, there are limitations to the size of the valve that can be implanted and there is no opportunity to repair additional defects such as pulmonary artery dilatation, which is frequently associated with severe PVR.

The advent of the hybrid surgical options for treating cardiac disease has integrated the techniques of interventional cardiology with the techniques of cardiac surgery to provide a form of therapy that combines the respective strengths of both fields.

In this focused review, we present and compare recent advances in traditional surgery, and hybrid procedures to replace the pulmonary valve in patients with ToF presenting with severe PVR and dilated right ventricular outlet tract (RVOT). We summarize the advantages and disadvantages of each approach and discuss the direction future research should take to determine the optimal management for individual patients.

## Surgical Outcomes

The surgical options for PVR are still regarded by many cardiologists and cardiac surgeons the “gold standard” for young adult patients with pulmonary regurgitation after their initial ToF repair. There are several recent studies describing long-term outcomes for surgical pulmonary valve replacement (SPVR). McKenzie et al. (4) conducted a retrospective analysis of outcomes in patients who underwent SPVR to provide a benchmark for comparison with the new emerging transcatheter techniques. Over a 15-year period, 247 patients with obstructive right heart lesions who developed pulmonary valve dysfunction were included in the study. More than 100 (42%) patients had a history of repaired ToF, and in 50% of the cases, regurgitation was the indication for SPVR. They showed average hospital stay of 5 days, 99% survival, and 94% freedom from reintervention at 5 years.

Sabate Rotes et al. (5) looked specifically at SPVR in patients with repaired ToF who develop PR, in a retrospective analysis of 278 cases over a 40-year period. They showed an early survival of 98.6% and 80% at 15 years, with a 97% freedom from pulmonary valve reintervention at 5 years. Patients who had undergone ToF repair at an older age had increased mortality, but also an increased protection against pulmonary valve reintervention.

A further large retrospective study of patients with a history of repaired ToF undergoing SPVR was undertaken by Babu-Narayan et al. (2). Over a 17-year period, 220 cases of SPVR were included. The group showed similar results to those of the studies previously mentioned with 97.7% 30-day survival, 96% survival at 3 years, and 96% freedom from reintervention at 10 years. Survival in the later era (2005–2010; *n* = 156) was significantly better compared with survival in the earlier era (1993–2004; *n* = 65; 99% vs 94% at 1 year and 98% vs 92% at 3 years, respectively; *p* = 0.019).

Table 1 (2, 4, 6–10) shows the results of the most recent surgical series with comparable study populations. It is quite clear from all these studies that there are large bodies of evidence to suggest that SPVR is a safe and effective technique in patients who develop PVR after repair of ToF. Patients therefore should be educated that SPVR does not confer higher procedural risk and has low in-hospital and midterm follow-up mortality and re-operation rates. Nevertheless, there is still an operative mortality risk that varies from 1% to almost 3%, and morbidity associated to the use of CPB and myocardial cardioplegic arrest. This will inevitably trigger a widescale inflammatory response with negative effects on several organ functions. Furthermore, there is a much higher chance of requiring blood product transfusion when CPB is used for corrective cardiac surgery. Another aspect to consider is that these reports all present the medium-term outcomes for homograft or allograft valves, which are chosen to avoid anti-coagulation in a cohort of young patients. While they ensure freedom from anti-coagulation, their durability is limited and over time the majority will degenerate and require re-operation. There have been several recent publications looking at using mechanical valves for PVR to try and reduce the rate of reintervention (11–15). One of the most serious complications with these valves is thrombosis although many thrombosed valves can be successfully treated with fibrinolytic therapy without needing valve extraction. The risk of bleeding events was low in these series. Mechanical valves may offer a longer term solution in a select group of patients who are compliant with anti-coagulation therapy or have already received a mechanical prosthesis on the tricuspid or the left side valves. Overall, these outcomes provide a useful benchmark for treatment strategy comparisons.

**Table 1:** **Outcomes comparison for recent published surgical pulmonary valve replacement series**.

First author	Year	Sample (*n*)	30-day mortality	5-year mortality	5-year redo-intervention
Babu-Narayan et al. (2)	2014	220	2%	4%	2%
McKenzie et al. (4)	2014	148	0	N/A	6%
Batlivala et al. (6)	2012	254	1.2%	1.9%	3%
Chen et al. (7)	2012	227	0	3%	6%
Lee et al. (8)	2012	170	1.2%	1.2%	2.9%
Jang et al. (9)	2012	131	0	0	3.5%
Zubairi et al. (10)	2011	169	0.6%	N/A	7%

## Percutaneous PVR

In 2000, pioneering work was carried out by Bonhoeffer et al. demonstrating the feasibility of percutaneous PVR (PPVR) in a 12–year-old patient (16). This minimally invasive approach offered the benefit of reducing the need for repeated open-valve replacement and potentially avoiding it all together.

Many groups have continued to develop the technique and shown its potential as a valuable alternative to SPVR. In the first extended human study of PPVR, Khambadkone et al. (17) showed successful valve deployment in 58 out of the 59 patients, 0% mortality at 10 months, but device complications in 14 patients (25%). However, these results have improved over time and Lurz et al. showed a reduction in complications to 2.5% (18).

Furthermore, the US Melody Valve Trial (19), which included 129 patients with dysfunctional RV-PA conduits was associated with 94% freedom from PV intervention at 1 year. Eicken et al. (20) produced a report lending further evidence to the feasibility of PPVR. This two-center study took 102 cases of PPVR as a treatment for pulmonary regurgitation in patients with dysfunctional RV to PA conduits. They were able to show a significant reduction in pulmonary regurgitation in all patients with a range of hemodynamic changes pointing to improved RV function postprocedure. However, they also showed stent fracture in 5% of the patients (a reduction from previously reported stent fractures rates of over 20%) and 8% of patients require repeat dilatation of the valve so highlighting the room for improvement with the technique. In an extension of their US Melody Valve Trial, McElhinney et al. (21) carried out some further observations to better understand the risk factors for these common complications in PPVR. In a multicenter study, they showed that patients with severely obstructed RVOT conduits were more likely to suffer stent fracture, and the benefit of pre-stenting in preventing stent fracture and reducing the incidence of reintervention.

Despite the rapid progress of PPVR, long-term outcomes as described for SPVR have not yet been produced. Recently, Dilber et al. (22) conducted a single-center retrospective study of patients with RVOT dysfunction who had undergone either SPVR or PPVR based on the indications for each of these procedures. The authors concluded that patients with RVOT dysfunction can be treated effectively using a complementary treatment concept, including SPVR and PPVR. This approach is dependent on decisions made by a team of surgeons and interventionists regarding which valve replacement techniques are appropriate for the patient in question. The PPVR patients were shown to benefit from shorter hospital stay, less complications postprocedure, and were more likely to return to usual daily activities sooner following discharge.

However, with the percutaneous technique, there is a limitation of the size of prosthesis, which can be inserted, currently up to 22 mm with the Melody valve (Medtronic Inc., Minneapolis, MN, USA) and up to 29 mm diameter with the Sapien transcatheter heart valve (Edwards Life-sciences LLC, Irvine, CA, USA) although off label uses of both have extended slightly beyond these measurement limitations (23–25). Therefore, in Dilber’s centre, which uses Melody valves (Medtronic Inc., Minneapolis, MN, USA), patients needed to have RVOT diameter <22 mm. Those patients with large RVOTs who often presented with pulmonary regurgitation automatically fell into the surgical group.

Moreover, the technique does not offer the opportunity of treating additional defects that are frequently associated with severe PR, such as pulmonary artery dilatation, and it cannot be used in the dilated native RVOT, which constitutes over 85% of patients with ToF requiring pulmonary valve replacement, because it requires a relative constriction for adequate fixation.

This highlights the main limitation of PPVR: it is not currently suitable for use in patients with native and large RVOTs, although some valve designs are evolving for use in the native RVOT they are unlikely to be widely clinically available anytime soon (26, 27). Boudjemline et al. (28) demonstrated an interesting technique in patients with large patched RVOTs to assess the feasibility of this technique as a treatment for pulmonary regurgitation in this patient group. The technique involved preparation of the large RVOTs with telescoping of multiple stents from the branch pulmonary arteries to act as a landing zone for concomitant transcatheter PV insertion. They showed 100% successful valve deployment, with no cases of stent fracture or migration. However, only 12 patients were included in this study, so a larger study with longer term follow-up would be needed to draw any firm conclusions.

As longer term outcomes are reported, there are also concerning reports of a higher incidence of bacterial endocarditis in PPVR when compared to the open approach. Buber et al. (29) reported a 9.5% incidence of bloodstream infections and 2.7% of patients had prothestic endocarditis after a median follow-up of 19 months. Reviewing results from the Melody Valve Trial, which included 311 patients with a median of 2.5 years follow-up, McElhinney et al. (30) found an annualized rate of transcatheter valve endocarditis of 0.88% per patient-year. When valve related endocarditis occurs, the majority of patients requires surgical prosthesis replacement (31). A recent comparison of the Melody valve with surgical implants demonstrated an incidence of Melody endocarditis of 7.5% during a median follow-up of 2 years (32). Homograft endocarditis rate was 2.4%, and endocarditis with the Contegra conduit was 20.4%. Survival free of endocarditis by Kaplan–Meier for homografts was 98.7% at 5 years; however, for the Melody valve, it was 84.9% and this is probably the most concerning longer term issue surrounding this valve.

## Hybrid PVR

Transcatheter pulmonary valve implantation has the potential to become the standard procedure in the treatment of dysfunctional conduits. Nevertheless, the majority of ToF patients (85%) presents with native outflow tracts and free pulmonary regurgitation and represents a big challenge for these new technologies. Over the last decade, there has been a significant evolution of cardiac surgery toward less invasive strategies, and at the same time, an expansion of interventional cardiology toward more complex procedures involving structural, congenital, and large vessel intervention. The development of hybrid cardiac catheterization laboratories has allowed a strong collaboration between surgeons and interventionalists mainly in the field of valve replacement, one of the fastest growing procedures in the United States.

**Figure 1:**
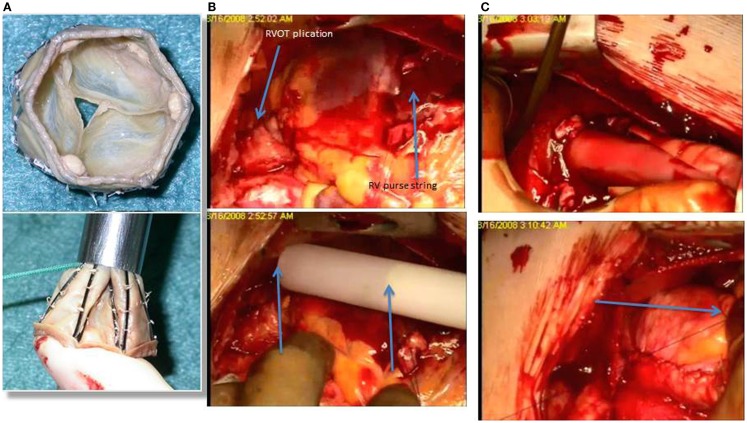
**(A)** The biopulmonic valve inserted into the delivery system; **(B)**, the RVOT has been plicated and a purse string passed in the anterior wall of the right ventricle, just below the RVOT; the introducer is then positioned on top of the RVOT and the length of the valve (arrows) is then compared with the length of the RVOT before the injection; **(C)** the biopulmonic valve within the delivery system is inserted into the RVOT through an incision below the previous transannular patch, and after delivery, the valve is stitched from the outside (arrow) to prevent any movement and possible migration.

At the Bristol Heart Institute and at the Rush University Medical Center, we have been pioneering new hybrid techniques for pulmonary valve replacement in ToF patients and we have recently published our first experience (33) with a newly developed tissue valve mounted on a self-expanding stent, the No-React Injectable BioPulmonic (BioIntegral Surgical Inc., Toronto, Canada) (Figure 1A). This technique requires only minimal mobilization of the heart and great vessels and thus reduces both the operative time and the risks associated with extensive dissection, such as bleeding and injury to the heart, great vessels, and adjacent structures (e.g., phrenic nerves). Furthermore, the valve is implanted without using CPB and cardioplegic arrest, thereby avoiding its adverse side effects. The Injectable BioPulmonic prosthesis currently is available in sizes up to a diameter of 31 mm.

This strategy requires a full median sternotomy, plication of the pulmonary artery and a stab incision on the anterior surface of the proximal RVOT just proximal to the infundibulum/infundibular patch, avoiding calcified tissue. A valve 2 mm in diameter larger than the maximum size measured is gently compressed into the introducer and slid into the provided trocar. The injector is then slid into the RVOT and advanced to the main PA (Figures 1B,C). The trocar delivery system is then withdrawn and the purse-string sutures controlled. Transesophageal and epicardial echocardiogram are used to assess the valve position throughout the process of deployment. The valve can then secured with external Prolene sutures placed in the proximal and distal rim of the valve. These pilot data on 10 patients comparing the off-pump hybrid pulmonary valve replacement (HPVR) with the on-pump conventional SPVR showed that off-pump HPVR was associated with a significant reduction in operating time (165.7 ± 43.3 range 110–240 min, vs 298.6 ± 57.4 range 221–375 min, *p* < 0.001) blood loss (83.3 ± 28.6 mL vs 527.1 ± 485.3 mL, *p* < 0.05), blood products requirement (0 units vs 3.6 ± 3.9 units, *p* < 0.05) and higher postoperative hemoglobin levels (13.4 ± 1.7 g/dL vs 9.8 ± 1.7 g/dL, *p* < 0.001) compared with on-pump SPVR. No patient had paravalvular leak or more than mild pulmonary regurgitation at an early follow-up, indicating that off-pump HPVR is a safe and effective surgical strategy and a possible beneficial alternative to the traditional on-pump PVR. A possible disadvantage of this technique is the fact that it still requires a significant incision into the anterior wall of the right ventricle, with possible early complications such as muscle tear and significant bleeding and long-term localized wall motion abnormalities and arrhythmias, even though we have not seen this complication in our patient population. Furthermore, there is potential risk of incorrect positioning of the valve either migrating toward the branch pulmonary arteries or sitting too proximally in the RVOT.

Dittrich et al. (34) reported a case of HPVR in a patient with enlarged RVOT after ToF repair. The technique employed was similar to ours, with the exception that access to the PA was via the third intercostal space and not a median sternotomy, and the valve was inserted through the pulmonary artery and not the right ventricle. They concluded that this technique was better than percutaneous transcatheter PVR in terms of its suitability for patients with enlarged RVOTs.

**Figure 2:**
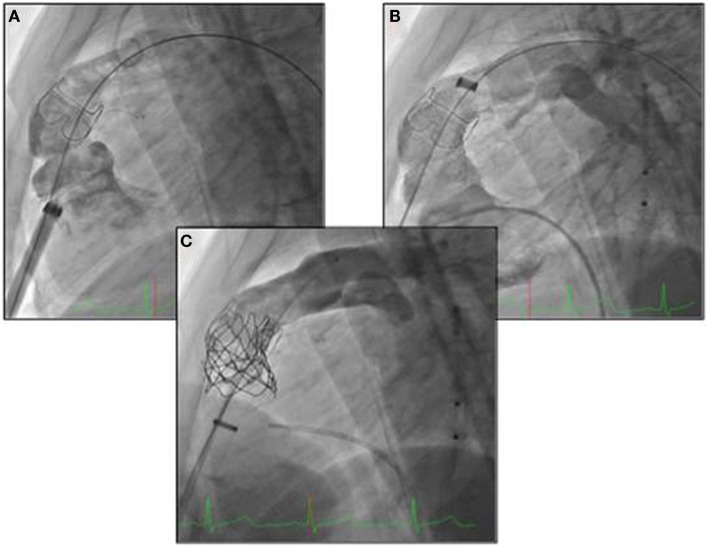
**(A, B)** Subxyphoid incision and a delivery sheath to implant the valve, inserted into the right ventricle; **(C)** delivery of the melody valve and controlled angiogram.

For small children with ToF and significant PR at the Rush University Medical Center, we have adopted a different hybrid technique for pulmonary valve implantation (35). The approach involved a subxyphoid incision and a delivery sheath to implant the valve, inserted into the right ventricle (Figure 2). This strategy has the advantage of avoiding a sternotomy (only the lower cartilaginous part of the sternum is divided leaving the main body of the bone intact) but still requires an opening of the right ventricle for the insertion of the valve (Figure 2).

**Figure 3:**
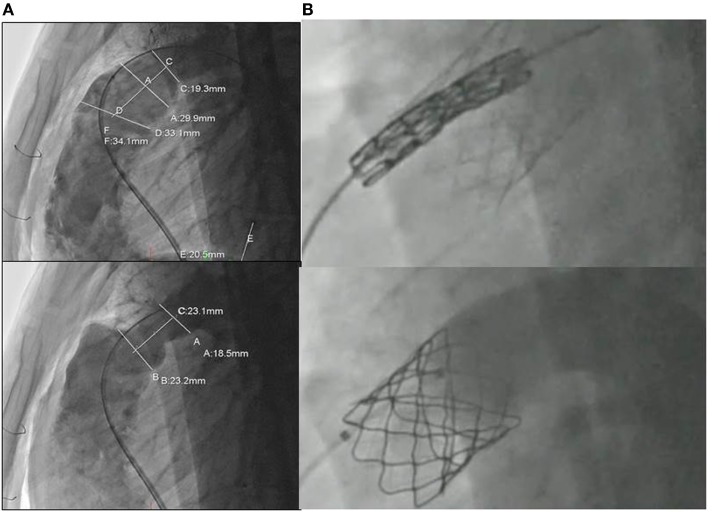
**(A)** Assessment and measurement of the RVOT with angiography before and after extensive plication of the previous transannular patch; **(B)** transcatheter insertion of the melody valve into the RVOT.

For the majority of patients with native RVOT and free PR post ToF repair, we have routinely adopted a hybrid strategy of pulmonary artery plication through a limited sternotomy or thoracotomy and transvenous insertion of a Melody pulmonary valve (36). The strategy is based on a preoperative angiogram for the assessment and measurement of the RVOT (Figure 3A). The pulmonary artery is then plicated in order to reduce its diameter by ~30% (Figure 3A). This creates a tubular structure that acts as a landing zone for the Melody valve (Figure 3B). A similar technique has been recently published as a case report by Travelli et al. (37), but instead of using a longitudinal plication, they applied a band around the main pulmonary artery. Postoperative angiography and intracardiac echocardiography are used to evaluate the efficacy of the treatment (Figure 3B). Our pilot data on the first five young adults who have received this technique is very encouraging, demonstrating an excellent quality of postoperative recovery and early discharge. Extensive calcification of the pre-existing RVOT patch may impede upon the ability to perform adequate placation, and this should be assessed with a pre-procedural postero-anterior and lateral chest X-ray. Use of a compliant sizing balloon to guide the extent of plication and to ensure there is an adequate-diameter landing zone following plication may help. Ensuring adequate stability of the pre-stent is a key, and a surgical suture to anchor the stent to the RVOT may be employed. Another limitation for the hybrid approach is the inability to perform associated surgical procedures such as right ventricular remodeling and RVOT ablation, even though the RVOT plication does significantly decrease the RVOT size. The presence of branch pulmonary artery stenosis can still be approached hybridly by stenting the vessels before the surgical RVOT plication and valve delivery. Nevertheless, branch PA narrowing requiring stenting may complicate the RVOT interventions particularly when there is left-sided pulmonary artery narrowing (usually provides the best site for the guidewire for pulmonary valve implantation). The carrot of the Melody valve may not traverse the stented vessel well, and this may impact upon stability of either the left pulmonary artery stent or the RVOT stent during valve implantation.

We have elected to deliver the valve from the groin rather than through the ventricle in an attempt to minimize myocardial scarring known to be a risk factor for ventricular tachyarrhythmia in this group of patients. Indeed, it is our attention to these marginal potential benefits that will define our successes with congenital heart disease patients over the next 30 years as mortality no longer becomes an acceptable outcome variable to define success. It is also possible that valve longevity in the absence of significant RVOT narrowing, shown to be a risk factor for valve stent degeneration (21) may be greater with this approach and certainly subjective assessment of external compressive forces on the valve stent in our early experience is encouraging. A les- invasive strategy for pulmonary artery plication using a thoracoscopic or a small thoracotomy approach should be considered in the future for further decreasing the invasiveness of this innovative hybrid technique, even though it is well proven that previous sternotomy does not increase the risk in congenital cardiac surgery (38). Some may question the validity of such an approach as at present it still requires a sternotomy. Indeed, the ideal solution is a transcatheter valve system designed specifically for the native outflow. Although two such systems are being evaluated in clinical trials, it may be many years before this technology is available, and therefore, we feel we should continue to push the boundaries for these patients, providing the least invasive options with optimal longer term outcomes including risk of arrhythmia and sudden cardiac death from myocardial scarring.

In our overall series of more than 20 patients who underwent an HPVR, at short-term follow-up (<4 years), all patients are alive and asymptomatic, with no signs of valve degeneration or infection. Longer follow-up, bigger sample size, and controlled randomized trials are required to clearly demonstrate the efficacy of the HPVR strategy compared with the SPVR, which is still considered the gold-standard procedure for patients with ToF and PVR.

## Conclusion

With the advance of less invasive technologies and new imaging techniques, a dedicated collaboration between cardiothoracic surgeons and interventionalists becomes mandatory for the treatment of congenital heart disease. Hybrid PVR is gaining increasing recognition as a less invasive alternative to SPVR, to minimize procedure time, risks and complications and greatly improve the quality of recovery for this population. These benefits to patients are expected to translate into benefits for the Health System as well, with fewer resources needed during the operation and for postoperative care. However, high-quality evidence, which can only be obtained by carrying out a randomized controlled trial, is needed to estimate the effectiveness and cost-effectiveness of the new technology of HPVR compared to the standard SPVR and to inform decisions about adoption of this interesting approach to RVOT dysfunction by surgeons and health care policy makers.

## Conflict of Interest Statement

The authors declare that the research was conducted in the absence of any commercial or financial relationships that could be construed as a potential conflict of interest.
